# Development and internal and external validation of a nomogram model for predicting the risk of chronic kidney disease progression in IgA nephropathy patients

**DOI:** 10.7717/peerj.18416

**Published:** 2024-10-30

**Authors:** Ying Zhang, Zhixin Wang, Wenwu Tang, Xinzhu Yuan, Xisheng Xie

**Affiliations:** 1 Department of Nephrology, Nanchong Central Hospital Affiliated to North Sichuan Medical College, Nanchong, Sichuan, China; 2 Department of Nephrology, Guangyuan Central Hospital, Guangyuan, Sichuan, China; 3 Nanchong Key Laboratory of Basic and Clinical Research of Chronic Kidney Disease, Nanchong, Sichuan, China; 4 Nanchong Clinical Medical Research Center, Nanchong, Sichuan, China

**Keywords:** IgA nephropathy, Chronic kidney disease, Risk of progress, Nomogram, External validation

## Abstract

**Background:**

IgA nephropathy (IgAN) is the most common primary glomerular disease in chronic kidney disease (CKD), exhibiting significant heterogeneity in both clinical and pathological presentations. We aimed to explore the risk factors influencing short-term prognosis (≥90 days) and to construct a nomogram model for evaluating the risk of CKD progression in IgAN patients.

**Methods:**

Clinical and pathological data of patients diagnosed with IgAN through biopsy at two centers were retrospectively collected. Logistic regression was employed to analyze the training cohort dataset and identify the independent predictors to construct a nomogram model based on the final variables. The predictive model was validated both internally and externally, with its performance assessed using the area under the curve (AUC), calibration curves, and decision curve analysis.

**Results:**

Out of the patients in the modeling group, 129 individuals (41.6%) did not achieve remission following 3 months of treatment, indicating a high risk of CKD progression. A multivariate logistic regression analysis demonstrated that body mass index, urinary protein excretion, and tubular atrophy/interstitial fibrosis were identified as independent predictors for risk stratification. A nomogram model was formulated utilizing the final variables. The AUCs for the training set, internal validation set, and external validation set were 0.746 (95% confidence intervals (CI) [0.691–0.8]), 0.764 (95% CI [0.68–0.85]), and 0.749 (95% CI [0.65–0.85]), respectively. The validation of the subgroup analysis also demonstrated a satisfactory AUC.

**Conclusion:**

This study developed and validated a practical nomogram that can individually predict short-term treatment outcomes (≥90 days) and the risk of CKD progression in IgAN patients. It provides reliable guidance for timely and personalized intervention and treatment strategies.

## Introduction

IgA nephropathy (IgAN) is the most common pathological form of primary glomerulonephritis, which is predominantly found in children and young adults. It constitutes 45–60% of primary glomerular diseases and ranks among the primary causes of end-stage renal disease (ESRD) in China (Hou et al., 2018; Stamellou et al., 2023). Based on reliable statistics, approximately 30% to 40% of patients experience progression to end-stage renal disease 20 to 30 years after the initial onset of clinical symptoms (Stamellou et al., 2023). These patients require renal replacement therapy to prolong their lives, which imposes a great physical, mental, and economic burden on the patients and their families and has a serious impact on the social economy (Lerma et al., 2023). A systematic review related to outcome prediction in IgAN revealed that renal dysfunction, dialysis, and mortality have consistently been focal points of concern. Factors such as age of onset, obesity, hypertension, proteinuria, and the degree of histological pathology all contribute to the progression of IgAN (Cattran, Floege & Coppo, 2023). Unfortunately, few studies have focused on the short-term prognosis (≥90 days) of IgAN, although it may ultimately affect long-term outcomes.

Early disease progression risk prediction and stratification remain great challenges among treatment decisions for IgAN patients. Proteinuria stands out as the most crucial independent predictor of adverse renal outcomes, serving as a dependable surrogate endpoint and therapeutic target for forecasting long-term clinical consequences (Thompson et al., 2019). The 2021 Guidelines of the Kidney Disease: Improving Global Outcomes Committee (KDIGO) (Beck et al., 2023) recommend the following treatment objectives for IgAN: reducing urinary protein to below 1 g/d for at least 90 days with optimized supportive therapy. In addition, risk prediction and stratification (high risk, >0.75–1 g/d) for progression of chronic kidney disease (CKD) are recommended to guide the development of clinical treatment strategies. According to this authoritative clinical practice guideline, a critical time point has been identified: 90 days after diagnosis. This is because the treatment approach in subsequent stages will be determined based on the extent of urinary protein remission at this stage. For patients with urinary protein levels exceeding 1 g/day, maintenance or intensification of immunosuppressive therapy is recommended. However, it is important to acknowledge that this situation may expose patients to a higher risk of adverse drug reactions and disease progression. In clinical practice, a significant number of patients still fail to achieve remission of proteinuria (>1 g/d) at least 90 days after treatment (>1 g/d), requiring maintenance or intensification of therapy. This situation increases the risk of adverse drug reactions and disease advancement. Therefore, early detection and management are crucial in improving early outcomes and preventing disease progression.

In this study, the objective was to retrospectively assess clinicopathological data and identify risk factors correlated with the chronic progression of IgAN. Additionally, we aimed to develop a nomogram model to identify the high-risk group among IgAN patients at an early stage and facilitate the formulation of individualized treatment regimens, ultimately reducing the risk of poor renal prognosis.

## Participants and Methods

### Participants

The primary study cohort comprised data from 443 primary IgAN patients who underwent kidney biopsies at Nanchong Central Hospital from January 1, 2015, to December 31, 2023. This data was utilized for model development and internal validation. In addition, data from 110 primary IgAN patients who underwent kidney biopsies at Guangyuan Central Hospital within the same healthcare system between January 1, 2024, and May 31, 2024, were used for external validation. The study adhered to the principles outlined in the Declaration of Helsinki and received approval from the Ethics Committee of two centers. Oral informed consent was obtained from each patient; however, written consent was waived due to the retrospective nature of the study (No: 2023-100).

**Figure 1:**
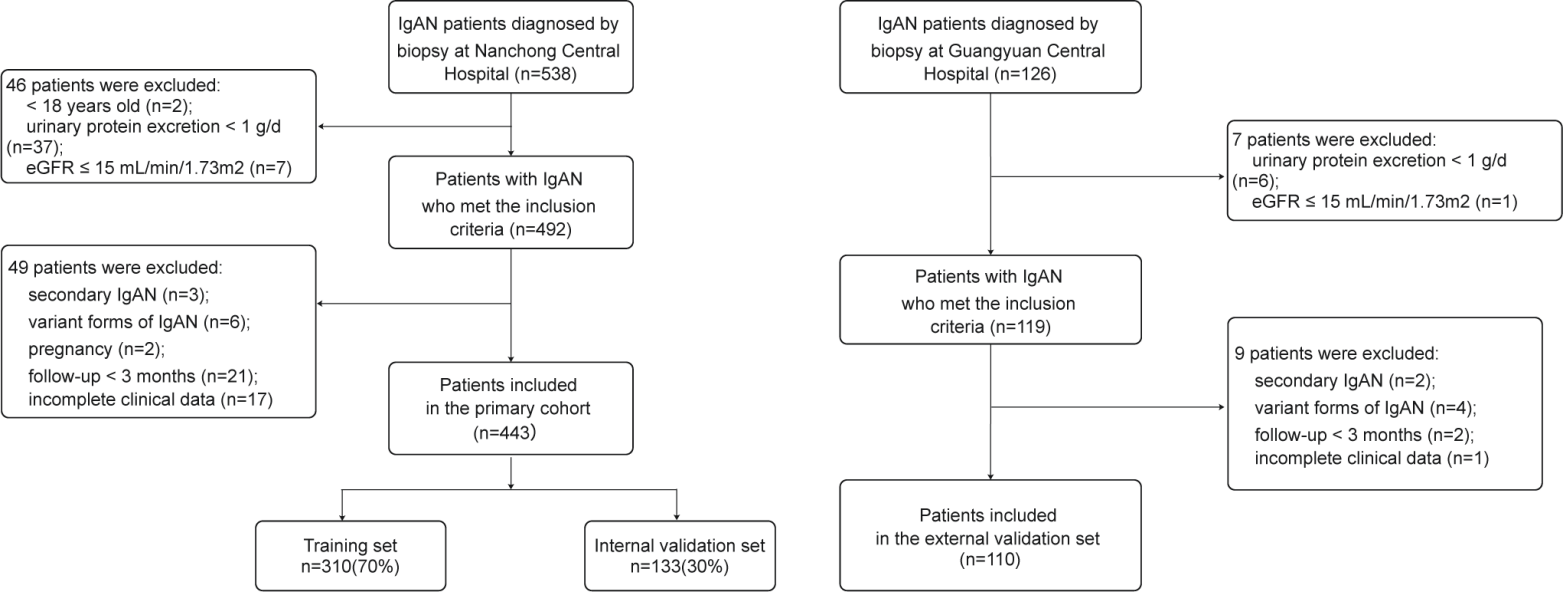
Recruitment process flowchart for research participants. IgAN, IgA nephropathy.

The inclusion and exclusion criteria were as follows. Inclusion criteria: (1) Age ≥ 18 years; (2) confirmed initial diagnosis of IgAN by renal biopsy; (3) urinary protein excretion (UPE) ≥ 1 g/d with estimated glomerular filtration rate (eGFR) >15 mL/min/1.73 m^2^. The exclusion criteria were as follows: (1) Secondary IgAN, including lupus nephritis, allergic purpura nephritis, and hepatitis B virus-associated glomerulonephritis; (2) variant forms of IgAN, including IgA deposition with minimal change disease, acute kidney injury, and rapidly progressive glomerulonephritis; (3) pregnancy; (4) follow-up for <3 months and incomplete clinical data (Fig. 1).

### Outcome and variables

The outcome variable in this study was the stratification of risk for progression of CKD. Here, we defined the outcome variable according to the 2021 KDIGO guidelines (Beck et al., 2023) and as outlined in the study by Canney et al. (2021). Low risk of CKD progression was defined as urinary protein <1 g/d and a decrease of at least 25% from baseline after renal biopsy and treatment for 90 days. High risk of CKD progression was defined as urinary protein ≥ 1 g/d after renal biopsy and treatment for 90 days, or a decrease in urinary protein less than 25% from baseline.

This was a cross-sectional survey that utilized baseline information from hospitalization records at the time of biopsy, and obtained through an electronic medical record system. The demographic and clinical data included sex, age, body mass index (BMI), systolic blood pressure (SBP), diastolic blood pressure (DBP), medical history, complications, and medication usage records. Laboratory data included neutrophil count, lymphocyte count, hemoglobin, platelet, serum albumin, serum creatinine, eGFR (calculated using the Chronic Kidney Disease Epidemiology Collaboration equation (Levey, Inker & Coresh, 2014)), serum uric acid, serum cystatin C, total cholesterol, triglycerides, serum high-sensitivity C-reactive protein (Hs-CRP), serum immunoglobulin and complement levels, and UPE. Renal biopsy data included Oxford Classification scores, which consist of mesangial hypercellularity (M): M0 ≤ 0.5, M1 > 0.5; endocapillary hypercellularity (E): E0 = absent, E1 = present; segmental glomerulosclerosis (S): S0 = absent, S1 = present; tubular atrophy/interstitial fibrosis (T): T0 < 25%, T1 = 25%–50%, T2 > 50%; and cellular/fibrocellular crescents (C): C0 = absent, C1 < 25%, C2 > 25% (Trimarchi et al., 2017). The histopathological findings of all renal biopsy specimens were independently evaluated by two pathology experts from the corresponding hospital. Decisions regarding supportive therapy and/or immunosuppressive treatment were made based on the judgment of the attending nephrology specialists.

### Development and validation of the nomogram

Before constructing the nomogram model, we randomly divided the patient data from the primary cohort into a training set and an internal validation set in a 7:3 ratio using R (dplyr 1.1.4). The nomogram was then developed using the training set data. Univariate logistic regression analysis and Forward Selection for Multivariate Logistic Regression were performed with SPSS 27.0 to identify independent predictors with statistical significance (*P* < 0.05). Subsequently, BMI, UPE, and T were used to construct the nomogram.

Internal and external validation were conducted on the nomogram. Model predictive accuracy was evaluated using 1,000 bootstrap resamples in R (rms 6.8.1). Receiver operating characteristic (ROC) curves were generated with R (pROC 1.18.5) to assess the model’s discriminative performance, while decision curve analysis (DCA) curves were created using R (rmda 1.6) to evaluate the model’s clinical utility. Additionally, we conducted a subgroup analysis based on different treatment regimens.

### Statistical analysis

Statistical analysis was executed utilizing SPSS 27.0, R 4.4.1. Continuous variables were assessed for normality using the Shapiro–Wilk test. Quantitative characteristics that were normally distributed were described as means and standard deviations, while those not normally distributed were reported as the median and interquartile range (IQR), and differences between groups were assessed through the Wilcoxon rank sum test. Qualitative characteristics were described as frequencies (percentages), and differences between groups were assessed through Pearson’s Chi-squared test or Fisher’s exact test. Logistic regression was conducted on the training set data to screen for key influencing factors, and the nomogram was generated using R software. The predictive capability of the model was assessed through the area under curve (AUC), calibration curve, and DCA. *P* < 0.05 was defined as a statistically significant difference.

## Results

### Baseline characteristics

With a rigorous inclusion and exclusion screening process, the study ultimately enrolled 553 primary IgAN patients, who were divided into 310 patients for the training set, 133 for the internal validation set, and 110 for the external validation set. Among them, 213 patients (38.5%) did not achieve remission of proteinuria at least 90 days of treatment, indicating a high risk for CKD progression. The study population predominantly consisted of young and middle-aged women, with an average age of 36 (28–48) years, and 56.2% of the patients were female. Specific baseline data for the study population are detailed in Table 1.

**Table 1:** Baseline characteristics of the study cohort.

Variables	Overall (*N* = 553)	Training set (*N* = 310)	Internal validation set (*N* = 133)	External validation set (*N* = 110)	*P* value
Non-remission, n (%)	213 (38.5)	129 (41.6)	46 (34.6)	38 (34.5)	0.240
Sex (male, %)	242 (43.8)	141 (45.5)	64 (48.1)	37 (33.6)	0.050
Age (years)	36.000 (28.000, 48.000)	35.000 (27.250, 47.000)	40.000 (28.000, 50.000)	38.000 (29.000, 46.000)	0.245
BMI (kg/m^2^)	23.880 (21.600, 26.000)	23.950 (21.405, 26.303)	24.030 (22.170, 26.080)	23.375 (20.848, 25.400)	0.073
SBP (mmHg)	131.000 (117.000, 144.000)	130.000 (115.000, 146.000)	132.500 (120.250, 144.750)	131.500 (118.250, 140.000)	0.448
DBP (mmHg)	85.000 (76.000, 95.000)	85.000 (76.000, 95.000)	86.000 (77.000, 95.750)	84.000 (75.250, 92.000)	0.560
Hypertension, n (%)					<0.001
NO	264 (48.7)	138 (45.7)	53 (40.8)	73 (66.4)	
Yes	278 (51.3)	164 (54.3)	77 (59.2)	37 (33.6)	
Diabetes, n (%)					0.020
NO	494 (89.3)	269 (86.8)	119 (89.5)	106 (96.4)	
Yes	59 (10.7)	41 (13.2)	14 (10.5)	4 (3.64)	
CKD stage, n (%)					0.004
Stage 1-2	439 (79.5)	238 (77.0)	101 (75.9)	100 (90.9)	
Stage 3-4	113 (20.5)	71 (23.0)	32 (24.1)	10 (9.09)	
Nephrotic syndrome, n (%)					0.306
NO	513 (92.8)	292 (94.2)	120 (90.2)	101 (91.8)	
Yes	40 (7.23)	18 (5.81)	13 (9.77)	9 (8.18)	
Neutrophil (×10^9^/L)	4.390 (3.440, 5.480)	4.440 (3.450, 5.810)	4.360 (3.300, 5.130)	4.370 (3.573, 5.445)	0.501
Lymphocyte (×10^9^/L)	1.590 (1.230, 2.000)	1.605 (1.240, 2.030)	1.670 (1.240, 2.010)	1.535 (1.193, 1.915)	0.413
Hemoglobin (g/L)	127.602 (21.606)	128.513 (21.595)	125.444 (21.544)	127.645 (21.725)	0.349
Platelet (×10^9^/L)	202.000 (157.000, 247.000)	209.500 (163.250, 255.750)	200.000 (157.000, 242.000)	185.500 (143.250, 223.500)	0.006
Albumin (g/L)	38.800 (35.400, 42.400)	38.700 (35.540, 42.575)	38.900 (35.300, 42.700)	39.300 (35.100, 41.675)	0.879
Serum creatinine (umol/L)	83.600 (63.950, 105.175)	84.000 (64.000, 108.600)	89.000 (65.000, 109.300)	76.500 (62.000, 94.000)	0.010
eGFR (ml/min/1.73 m2)	88.440 (65.163, 111.565)	88.540 (62.870, 112.180)	82.320 (61.870, 105.470)	97.025 (73.798, 115.843)	0.011
Serum uric acid (umol/L)	362.100 (297.925, 434.000)	362.500 (291.600, 440.000)	378.900 (319.600, 434.800)	348.000 (293.250, 414.250)	0.114
Serum cystatin C (mg/L)	1.100 (0.890, 1.470)	1.070 (0.883, 1.643)	1.140 (0.890, 1.590)	1.070 (0.940, 1.280)	0.416
Total cholesterol (mmol/L)	4.900 (4.190, 5.690)	4.890 (4.140, 5.810)	4.930 (4.213, 5.780)	4.870 (4.315, 5.235)	0.714
Triglycerides (mmol/L)	1.640 (1.090, 2.360)	1.560 (1.018, 2.293)	1.550 (1.075, 2.455)	1.750 (1.295, 2.278)	0.508
Hs-CRP (mg/L)	2.000 (0.650, 4.500)	2.125 (0.635, 4.860)	1.300 (0.600, 4.370)	2.165 (0.793, 3.980)	0.321
Serum IgG (g/L)	10.120 (8.060, 12.240)	9.985 (7.955, 12.100)	10.300 (7.840, 13.040)	10.460 (8.445, 12.178)	0.381
Serum IgM (g/L)	1.240 (0.890, 1.760)	1.180 (0.854, 1.717)	1.220 (0.870, 1.850)	1.360 (1.000, 1.740)	0.320
Serum IgA (g/L)	2.920 (2.340, 3.630)	2.995 (2.350, 3.728)	2.720 (2.200, 3.670)	2.910 (2.453, 3.403)	0.311
Serum C3 (g/L)	0.940 (0.810, 1.080)	0.903 (0.780, 1.050)	0.884 (0.798, 1.042)	1.045 (0.940, 1.150)	<0.001
Serum C4 (g/L)	0.260 (0.207, 0.326)	0.261 (0.204, 0.321)	0.254 (0.201, 0.327)	0.260 (0.224, 0.330)	0.587
UPE (g/d)	1.910 (1.430, 3.060)	1.950 (1.473, 3.143)	2.140 (1.660, 3.220)	1.445 (1.195, 2.190)	<0.001
Hematuria (RBCs/ul)	68.500 (12.000, 198.000)	88.000 (15.750, 252.250)	90.500 (17.750, 241.000)	16.000 (5.000, 69.000)	<0.001
**Oxford classification, n (%)**					
M1	536 (96.9)	297 (95.8)	129 (97.0)	110 (100.0)	0.064
E1	215 (38.9)	126 (40.6)	49 (36.8)	40 (36.4)	0.628
S1	310 (56.1)	202 (65.2)	75 (56.4)	33 (30.0)	<0.001
T1/2	181 (32.7)	106 (34.2)	49 (36.8)	26 (23.6)	0.065
C1/2	197 (35.6)	124 (40.0)	62 (46.6)	11 (10.0)	<0.001
**Treatment, n (%)**					0.753
RAASi alone	200 (36.2)	112 (36.1)	47 (35.3)	41 (37.3)	
RAASi + Glucocorticoid	175 (31.6)	100 (32.3)	37 (27.8)	38 (34.5)	
RAASi + Glucocorticoid + Immunosuppressant	60 (10.8)	30 (9.68)	18 (13.5)	12 (10.9)	
Others	118 (21.3)	68 (21.9)	31 (23.3)	19 (17.3)	

**Notes.**

Kruskal–Wallis rank sum test or Pearson’s Chi-squared test or Fisher’s exact test was used for comparison between groups.

BMI, body mass index; SBP, systolic blood pressure; DBP, diastolic blood pressure; CKD, chronic kidney disease; eGFR, estimated glomerular filtration rate; hs-CRP, high-sensitivity C-reactive protein; UPE, urinary protein excretion; RBC, red blood cells; M, mesangial hypercellularity; E, endocapillary hypercellularity; S, segmental glomerulosclerosis; T, interstitial fibrosis/tubular atrophy; C, crescent formation; RAASi, renin-angiotensin-aldosterone system inhibitors, including angiotensin converting enzyme inhibitor and angiotensin receptor blocker; Immunosuppressant included Cytoxan, mycophenolate mofetil, Cyclosporine, Tacrolimus.

In the training set, patients in the high-risk group exhibited significantly higher levels of BMI, DBP, neutrophil count, serum creatinine, serum cystatin C, cholesterol, triglycerides, hs-CRP, serum C3, UPE and hematuria compared to those in the low-risk group. Furthermore, the high-risk group demonstrated a higher prevalence of diabetes, CKD3-4 stage and nephrotic syndrome. They also displayed more severe renal tubular atrophy/interstitial fibrosis and greater formation of cellular/fibrocellular crescents. Meanwhile, the high-risk group had lower levels of albumin, eGFR and serum IgG (Table 2).

**Table 2:** Baseline characteristics of the training set.

Variables	Overall (*N* = 310)	Low risk (*N* = 181)	High risk (*N* = 129)	*P* value
Sex (male, %)	141 (45.5)	80 (44.2)	61 (47.3)	0.590
Age (years)	35.000 (27.250, 47.000)	34.000 (27.000, 46.000)	36.000 (28.000, 49.000)	0.177
BMI (kg/m^2^ )	23.950 (21.405, 26.303)	22.890 (20.700, 25.530)	24.910 (22.950, 27.480)	<0.001
SBP (mmHg)	130.000 (115.000, 146.000)	129.500 (114.750, 144.250)	131.000 (118.000, 146.000)	0.331
DBP (mmHg)	85.000 (76.000, 95.000)	83.000 (75.000, 93.250)	88.000 (79.000, 97.000)	0.013
Hypertension, n (%)				0.112
NO	138 (45.7)	89 (49.4)	49 (40.2)	
Yes	164 (54.3)	91 (50.6)	73 (59.8)	
Diabetes, n (%)				0.018
NO	269 (86.8)	164 (90.6)	105 (81.4)	
Yes	41 (13.2)	17 (9.39)	24 (18.6)	
CKD stage, n (%)				<0.001
Stage 1-2	238 (77.0)	152 (84.4)	86 (66.7)	
Stage 3-4	71 (23.0)	28 (15.6)	43 (33.3)	
Nephrotic syndrome, n (%)				<0.001
NO	292 (94.2)	179 (98.9)	113 (87.6)	
Yes	18 (5.81)	2 (1.10)	16 (12.4)	
Neutrophil (×10^9^/L)	4.440 (3.450, 5.810)	4.250 (3.360, 5.470)	4.690 (3.590, 6.470)	0.026
Lymphocyte (×10^9^/L)	1.605 (1.240, 2.030)	1.560 (1.220, 2.030)	1.610 (1.310, 2.030)	0.291
Hemoglobin (g/L)	128.513 (21.595)	128.895 (19.367)	127.977 (24.450)	0.756
Platelet (×10^9^/L)	209.500 (163.250, 255.750)	214.000 (168.000, 252.000)	205.000 (155.000, 263.000)	0.596
Albumin (g/L)	38.700 (35.540, 42.575)	39.900 (37.000, 43.300)	37.500 (33.500, 41.200)	<0.001
Serum creatinine (umol/L)	84.000 (64.000, 108.600)	79.450 (63.150, 103.550)	88.900 (67.000, 133.200)	0.028
eGFR (ml/min/1.73 m2)	88.540 (62.870, 112.180)	90.060 (72.403, 112.943)	85.560 (47.210, 110.490)	0.028
Serum Uric acid (umol/L)	362.500 (291.600, 440.000)	360.400 (288.100, 435.650)	368.600 (292.200, 443.700)	0.564
Serum cystatin C (mg/L)	1.070 (0.883, 1.643)	1.030 (0.820, 1.360)	1.140 (0.900, 1.920)	0.027
Total cholesterol (mmol/L)	4.890 (4.140, 5.810)	4.670 (4.030, 5.560)	5.315 (4.513, 6.028)	<0.001
Triglycerides (mmol/L)	1.560 (1.018, 2.293)	1.440 (0.990, 1.978)	1.805 (1.130, 2.873)	0.003
Hs-CRP (mg/L)	2.125 (0.635, 4.860)	1.210 (0.550, 4.450)	3.230 (0.870, 5.380)	0.022
Serum IgG (g/L)	9.985 (7.955, 12.100)	10.490 (8.460, 12.300)	9.300 (7.490, 11.500)	0.006
Serum IgM (g/L)	1.180 (0.854, 1.717)	1.270 (0.950, 1.700)	1.110 (0.780, 1.720)	0.147
Serum IgA (g/L)	2.995 (2.350, 3.728)	3.090 (2.430, 3.750)	2.760 (2.260, 3.660)	0.056
Serum C3 (g/L)	0.903 (0.780, 1.050)	0.888 (0.780, 1.020)	0.950 (0.792, 1.080)	0.034
Serum C4 (g/L)	0.261 (0.204, 0.321)	0.250 (0.200, 0.310)	0.270 (0.210, 0.346)	0.126
UPE (g/d)	1.950 (1.473, 3.143)	1.700 (1.380, 2.370)	2.780 (1.760, 5.010)	<0.001
Hematuria (RBCs/ul)	88.000 (15.750, 252.250)	73.500 (11.000, 180.000)	124.000 (52.250, 385.000)	0.002
**Oxford classification, n (%)**				
M1	297 (95.8)	173 (95.6)	124 (96.1)	0.814
E1	126 (40.6)	67 (37.0)	59 (45.7)	0.123
S1	202 (65.2)	116 (64.1)	86 (66.7)	0.639
T1/2	106 (34.2)	45 (24.9)	61 (47.3)	<0.001
C1/2	124 (40.0)	62 (34.3)	62 (48.1)	0.014
**Treatment, n (%)**				0.907
RAASi alone	112 (36.1)	66 (36.5)	46 (35.7)	
RAASi + Glucocorticoid	100 (32.3)	56 (30.9)	44 (34.1)	
RAASi + Glucocorticoid + Immunosuppressant	30 (9.68)	19 (10.5)	11 (8.53)	
Others	68 (21.9)	40 (22.1)	28 (21.7)	

**Notes.**

Wilcoxon rank sum test or Pearson’s Chi-squared test was used for comparison between groups.

BMI, body mass index; SBP, systolic blood pressure; DBP, diastolic blood pressure; CKD, chronic kidney disease; eGFR, estimated glomerular filtration rate; hs-CRP, high-sensitivity C-reactive protein; UPE, urinary protein excretion; RBC, red blood cells; M, mesangial hypercellularity; E, endocapillary hypercellularity; S, segmental glomerulosclerosis; T, interstitial fibrosis/tubular atrophy; C, crescent formation; RAASi, renin-angiotensin-aldosterone system inhibitors, including angiotensin converting enzyme inhibitor and angiotensin receptor blocker; Immunosuppressant included Cytoxan, mycophenolate mofetil, Cyclosporine, Tacrolimus.

### Identification of risk factors

Univariate logistic regression analysis was applied to analyze the baseline variables of the training set. The results showed that 17 variables, including BMI, DBP, neutrophil count, albumin, serum creatinine, eGFR, serum cystatin C, triglycerides, serum IgG, serum C3, UPE, hematuria, CKD stage, nephrotic syndrome, diabetes, T, and C, were statistically significant factors (*P* < 0.05). Multivariate logistic regression analysis was then executed on those characteristics (Table 3).

**Table 3:** Logistic regression assessing risk factors for CKD progression risk.

Variables	Univariable analysis				Multivariable analysis		
	*OR*	95%*CI*	*P* value		*OR*	95%*CI*	*P* value
Sex (male)	1.133	0.720–1.782	0.591				
Age (years)	1.013	0.995–1.03	0.151				
BMI (kg/m^2^)	1.103	1.04–1.169	0.001		1.109	1.042–1.182	0.001
SBP (mmHg)	1.004	0.993–1.014	0.472				
DBP (mmHg)	1.021	1.003–1.038	0.019				
Hypertension	1.457	0.915–2.320	0.113				
Diabetes	2.205	1.131–4.300	0.02				
CKD stage							
Stage 1–2	Ref	Ref	Ref				
Stage 3–4	2.603	1.517–4.469	0.001				
Nephrotic syndrome	12.673	2.860–56.155	0.001				
Neutrophil (×10^9^/L)	1.161	1.05–1.283	0.004				
Lymphocyte (×10^9^/L)	1.18	0.853–1.634	0.318				
Hemoglobin (g/L)	0.998	0.988–1.009	0.712				
Platelet (×10^9^/L)	1	0.997–1.003	0.925				
Albumin (g/L)	0.921	0.883–0.96	<0.001				
Serum creatinine (umol/L)	1.006	1.002–1.01	0.006				
eGFR (ml/min/1.73 m2)	0.991	0.984–0.998	0.014				
Serum Uric acid (umol/L)	1.001	0.999–1.003	0.556				
Serum cystatin C (mg/L)	1.616	1.15–2.271	0.006				
Total cholesterol (mmol/L)	1.326	1.113–1.579	0.002				
Triglycerides (mmol/L)	1.114	0.984–1.262	0.088				
Hs-CRP (mg/L)	0.994	0.974–1.015	0.567				
Serum IgG (g/L)	0.901	0.83–0.978	0.013				
Serum IgM (g/L)	0.92	0.672–1.26	0.603				
Serum IgA (g/L)	0.817	0.645–1.034	0.093				
Serum C3 (g/L)	5.155	1.557–17.067	0.007				
Serum C4 (g/L)	11.279	0.931–136.657	0.057				
UPE (g/d)	1.561	1.332–1.829	<0.001		1.502	1.268–1.779	<0.001
Hematuria (RBCs/ul)	1.001	1.0–1.001	0.032				
**Oxford classification**							
M1	1.147	0.366–3.589	0.814				
E1	1.434	0.906–2.270	0.124				
S1	1.121	0.697–1.803	0.639				
T1/2	2.711	1.673–4.394	<0.001		2.134	1.226–3.714	0.007
C1/2	1.776	1.119–2.819	0.015				
**Treatment**							
RAASi alone	0.996	0.540–1.837	0.989				
RAASi + Glucocorticoid	1.122	0.602–2.095	0.717				
RAASi + Glucocorticoid + Immunosuppressant	0.827	0.341–2.006	0.674				
Others	Ref	Ref	Ref				

**Notes.**

*OR*, odds ratio; *CI*, confidence interval; BMI, body mass index; SBP, systolic blood pressure; DBP, diastolic blood pressure; CKD, chronic kidney disease; eGFR, estimated glomerular filtration rate; hs-CRP, high-sensitivity C-reactive protein; UPE, urinary protein excretion; RBC, red blood cells; M, mesangial hypercellularity; E, endocapillary hypercellularity; S, segmental glomerulosclerosis; T, interstitial fibrosis/tubular atrophy; C, crescent formation. RAASi, renin-angiotensin-aldosterone system inhibitors, including angiotensin converting enzyme inhibitor and angiotensin receptor blocker; Immunosuppressant included Cytoxan, mycophenolate mofetil, Cyclosporine, Tacrolimus.

The multivariate logistic regression model identified three risk factors independently correlated with CKD progression risk: BMI (OR = 1.109, 95% CI [1.042–1.182], *P* = 0.001), UPE (OR = 1.502, 95% CI [1.268–1.779], *P* < 0.001), and T1/2 (OR = 2.134, 95% CI [1.226–3.714], *P* = 0.007). The remaining factors did not show significant statistical significance (*P* > 0.05, Table 3).

### Establishment and validation of the nomogram

**Figure 2:**
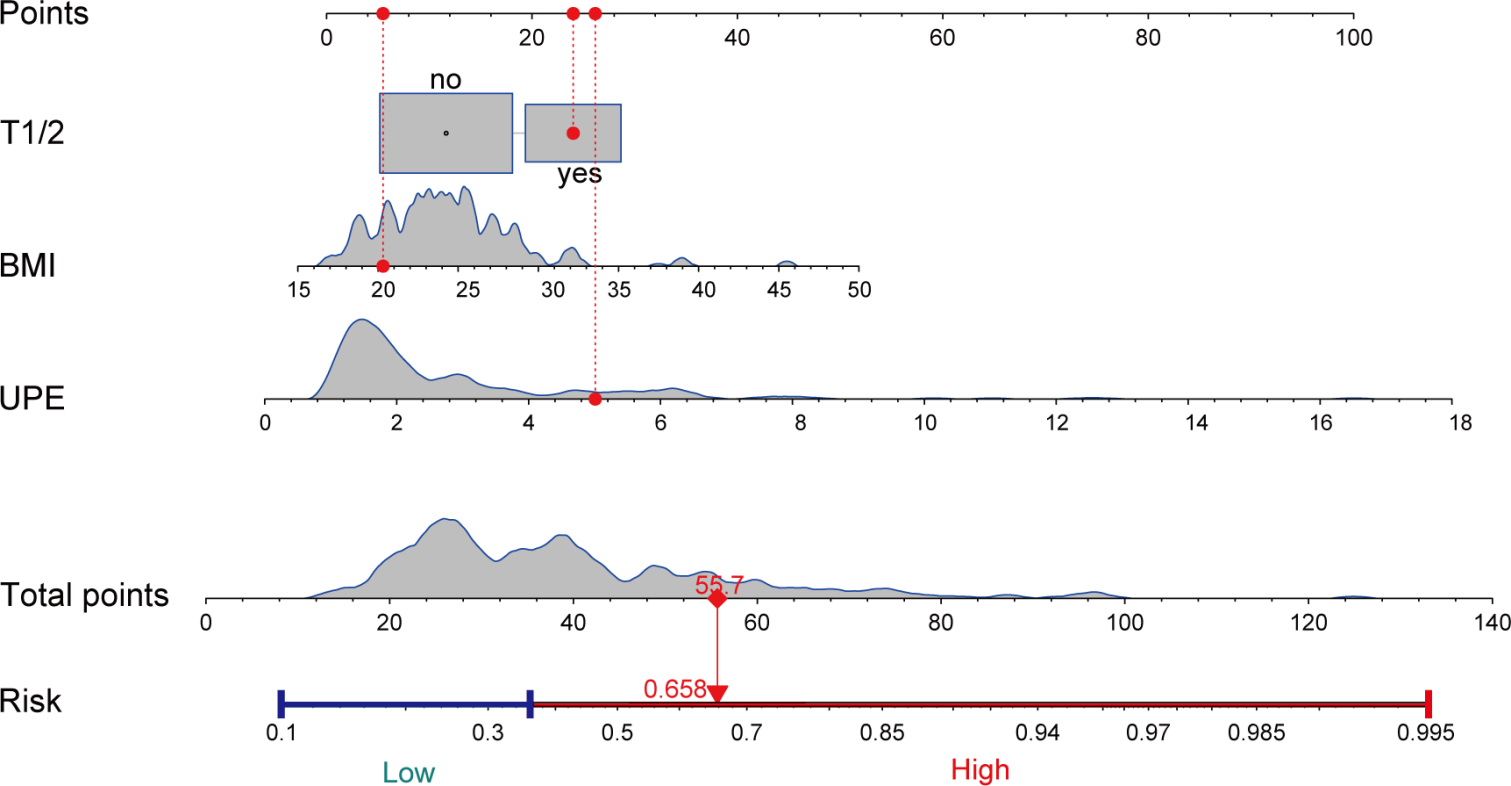
Nomogram to estimate the risk of CKD progression in IgAN patients. We visualized the application of the nomogram model based on clinical cases (highlighted in red). Each predictor in the case receives a corresponding “Points”, and summed to obtain a “Total points”, and plotting a vertical line downward yields a corresponding risk value to determine the risk of CKD progression. T, tubular atrophy/interstitial fibrosis; BMI, body mass index; UPE, urinary protein excretion.

A nomogram was created using the results of the multivariate logistic regression analysis, which identified three risk factors. This nomogram visualizes the practical use of the model using a random sample (Fig. 2). The nomogram model assigns scores to each independent variable based on their contribution to the outcome event (as indicated by the magnitude of regression coefficients). These scores are then summed to obtain a total score, which is converted into a probability score. Higher scores indicate a higher risk of CKD progression. An example of the nomogram applied to a random sample is illustrated in Fig. 2.

**Figure 3:**
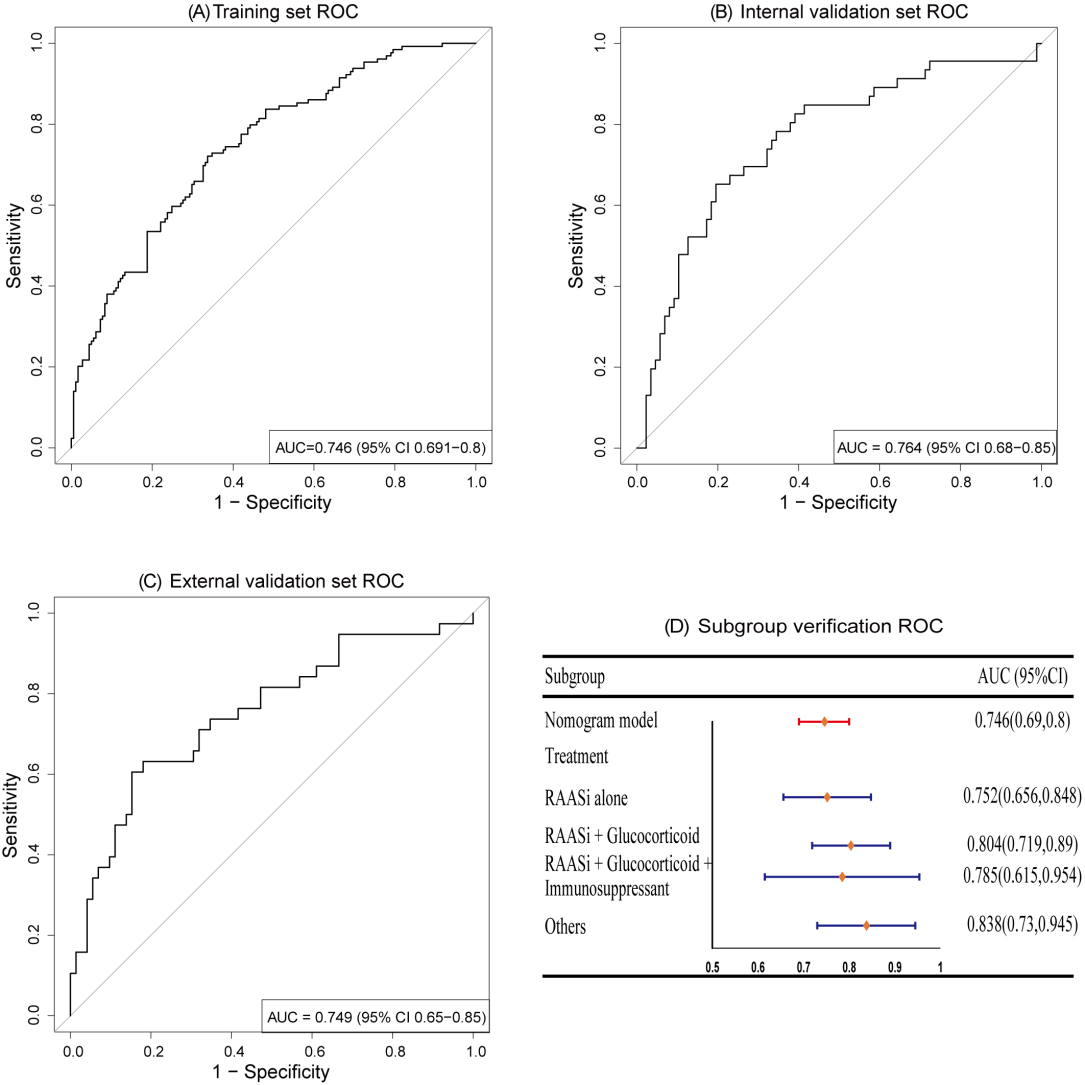
ROC curves. The AUCs of training set (A), internal validation set (B), and external validation set (C) showed that the model has a good discrimination ability. Subgroup validation of AUCs based on treatment regimens (D).

We employed R (pROC 1.18.5) and R (rms 6.8.1) to generate ROC curves and calibration curves for evaluating the predictive performance of the model on the training set and validation set. The results demonstrate that the nomogram model exhibits good discriminative ability, with AUCs of 0.746 (95% CI [0.691–0.8]), 0.764 (95% CI [0.68–0.85]), and 0.749 (95% CI [0.65–0.85]) for the training set, internal validation set, and external validation set, respectively; subgroup analysis validation based on treatment measures also showed a satisfied AUC, as depicted in Fig. 3.

**Figure 4:**
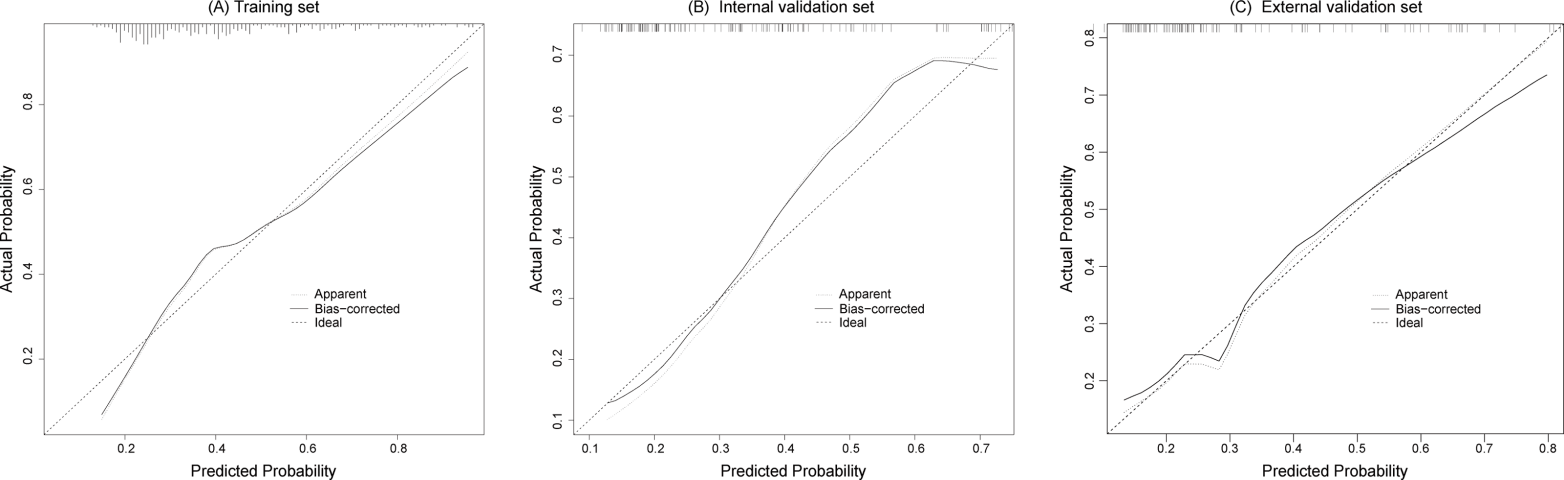
The calibration curves of the training set (A), internal validation set (B) and external validation set (C) both fit well with the ideal curve, demonstrating good consistency between predicted and actual risk probability.

The calibration curves fitted well with the ideal curve in the training set and the internal and external validation sets, indicating a high level of consistency between the predicted probabilities and actual event rates of the model, as shown in Fig. 4.

**Figure 5:**
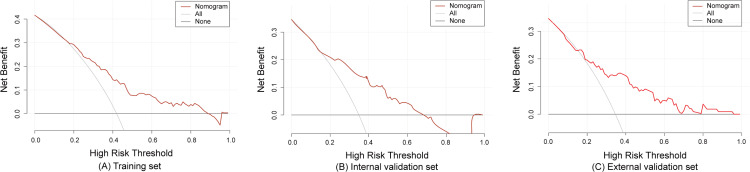
The decision curve analysis of the training set (A), internal validation set (B) and external validation set (C).

The model demonstrated good predictive accuracy in discrimination and calibration; however, its clinical utility remained uncertain. To validate the clinical applicability of the model, we plotted DCA curves using R (rmda 1.6). The DCA curves revealed that the net clinical benefit of the model decreased as the threshold probability increased. Specifically, when the threshold probability values ranged from 1.0% to 88.0% for the training set, and 1.0% to 68.0% for the internal validation set, and 1.0% to 95% for the external validation set, the model provided a net benefit that surpassed both “treat all” or “treat none” strategies, as depicted in Fig. 5.

## Discussion

Our study discovered that even with proactive treatment at least 90 days after diagnosis, 38.5% of IgAN patients did not experience remission in proteinuria, requiring the continuation or intensification of therapy. The finding indicates a high prevalence of patients at risk for CKD progression in clinical practice, highlighting the need for increased attention to this issue. Further univariate and multivariate logistic regression analysis indicates that the lack of reduction in proteinuria in these patients may be closely associated with BMI, UPE, and T1/2. Significantly, we developed a nomogram model based on these three variables and performed both internal and external validation. The results show that the nomogram model demonstrates good discriminative ability, predictive accuracy, and clinical utility in both the training set and the validation set.

BMI is the primary indicator of weight assessment in clinical practice worldwide. Yun et al. (2018) found that obesity, whether accompanied by metabolic disorders or not, can lead to progression and is an independent risk factor for the deterioration of CKD. Bonnet et al. (2001) first proposed in 2001 that obesity was a novel independent risk factor for the clinical and pathological progression of primary IgAN, which was subsequently corroborated by multiple studies (Berthoux, Mariat & Maillard, 2013; Shimamoto et al., 2015; Wu, Wang & Li, 2018; Kataoka et al., 2012). Recently, a meta-analysis has shown that higher BMI in IgAN patients might be associated with lower kidney function (Kanbay et al., 2022). IgAN patients complicated with obesity had more severe renal dysfunction at the time of renal biopsy than those with optimal body weight (Wang et al., 2023). In our research, we found that a high BMI is a risk factor for CKD progression in patients with IgAN. Notably, our study had a limited observation period of three months and minimal fluctuations in BMI, which enhances the reliability of our findings. Excessive weight can potentially trigger or exacerbate renal pathological damage by affecting intrarenal hemodynamics (increased renal blood flow and hyperfiltration), accelerating the progression of kidney disease in patients. Meanwhile, it was reported that high BMI indirectly accelerated the progression of IgAN by inducing metabolic syndrome (Kataoka et al., 2012). Furthermore, consistent with the findings of previous studies, our research also highlights baseline proteinuria as a significant risk factor influencing the progression of CKD in patients with IgAN (Gadola et al., 2023; Chen et al., 2018).

Several studies have explored the relationship between Oxford classification indicators and the prognosis of IgAN, with T1/2 holding the most significant prognostic value. The results of the VALIGA study, which followed 1,147 IgAN patients from 13 European countries to assess the predictive value of the Oxford classification for renal outcomes, revealed that T1/2 lesions serve as independent predictors of a 50% decrease in eGFR or ESRD (Coppo et al., 2014). This conclusion was similarly supported by the study after 4 years of updated follow-up (Coppo et al., 2020). Similarly, Chen, Wu & Tsai (2023) found that T1/2 was the most predictive variable for renal prognosis (AUC = 0.73), consistently correlating with poorer renal outcomes across all subgroups and baseline states. Our research also identified that T1/2 is a predictor of short-term prognosis and disease progression in patients with IgAN (Tang et al., 2023). Strikingly, a recent systematic evaluation indicated that T1/2 was the Oxford element most frequently associated with IgAN outcomes (Howie & Lalayiannis, 2023). Given that T1/2 scoring is more objective and exhibits better reproducibility, it largely reflects a chronic pathological state that cannot be reversed through treatment at the time of biopsy, as well as a higher impact of tubular injury on renal function compared to glomerular injury. This could explain the significant association between T-scoring and disease progression (Howie & Lalayiannis, 2023).

IgAN is the most frequent primary glomerular nephritis and one of the leading reasons of ESRD (Hou et al., 2018). While numerous risk models have been developed to predict the progression of IgAN, these models often focus on ESRD or eGFR decline as the observed endpoints, which tends to overlook the importance of short-term efficacy (Barbour et al., 2019; Schena et al., 2021). To the best of our knowledge, this is the first nomogram model to predict treatment response and risk of CKD progression after 90 days of diagnosis and treatment in patients with IgAN. Furthermore, it has been externally validated. However, there are some limitations to this study. Firstly, it was retrospective study, where all baseline data were derived from renal biopsy patients. This overlooks patients with milder conditions who did not undergo renal biopsy, which may introduce selection bias. Secondly, although our model has been externally validated, it is worth noting that the model was developed using a relatively small sample size and both the development cohort and validation cohort were from a single province in China, which may affect the generalizability and applicability of our findings to a broader population. Furthermore, this limited our full consideration of certain variables (*e.g.*, basement membrane thickness, degree of podocyte fusion, and proportion of glomerulosclerosis in the analysis of pathologic biopsies) due to the exposure to some missing data. These unconsidered variables may affect the accuracy of the outcome indicators. Therefore, future research should incorporate additional variables and larger sample sizes. Additionally, conducting prospective and multicenter study is necessary to further validate the factors identified in our analysis that influence disease progression in primary IgAN patients.

## Conclusion

In summary, this study found that IgAN patients with poor short-term efficacy (≥90 days and UPE ≥ 1 g/d) and a high risk of CKD progression accounted for a high proportion of the overall population. Through the nomogram predictive model, high-risk individuals for CKD progression among IgAN patients can be better identified, enabling early intervention to halt disease progression. These findings carry important implications for guiding personalized clinical interventions.

## Supplemental Information

10.7717/peerj.18416/supp-1 Supplemental Information 1 Raw data

10.7717/peerj.18416/supp-2 Supplemental Information 2 R codes and packages for drawing pictures

10.7717/peerj.18416/supp-3 Supplemental Information 3 Codebook

## Additional Information and Declarations

### Data Availability

The following information was supplied regarding data availability: The raw data and R code are available at GitHub and Zenodo: - https://github.com/zhangy-hub/Raw-data-and-code-files/tree/v1.0.0
- zhangy-hub. (2024). zhangy-hub/Raw-data-and-code-files: Raw data and code files (v1.0.0). Zenodo. https://doi.org/10.5281/zenodo.13624981.

## References

[ref-1] Barbour SJ, Coppo R, Zhang H, Liu Z-H, Suzuki Y, Matsuzaki K, Katafuchi R, Er L, Espino-Hernandez G, Kim SJ, Reich HN, Feehally J, Cattran DC (2019). Evaluating a new international risk-prediction tool in IgA nephropathy. JAMA Internal Medicine.

[ref-2] Beck LH, Ayoub I, Caster D, Choi MJ, Cobb J, Geetha D, Rheault MN, Wadhwani S, Yau T, Whittier WL (2023). KDOQI US commentary on the 2021 KDIGO clinical practice guideline for the management of glomerular diseases. American Journal of Kidney Diseases.

[ref-3] Berthoux F, Mariat C, Maillard N (2013). Overweight/obesity revisited as a predictive risk factor in primary IgA nephropathy. Nephrology, Dialysis, Transplantation.

[ref-4] Bonnet F, Deprele C, Sassolas A, Moulin P, Alamartine E, Berthezène F, Berthoux F (2001). Excessive body weight as a new independent risk factor for clinical and pathological progression in primary IgA nephritis. American Journal of Kidney Diseases.

[ref-5] Canney M, Barbour SJ, Zheng Y, Coppo R, Zhang H, Liu Z-H, Matsuzaki K, Suzuki Y, Katafuchi R, Reich HN, Cattran D (2021). Quantifying duration of proteinuria remission and association with clinical outcome in IgA nephropathy. Journal of the American Society of Nephrology.

[ref-6] Cattran DC, Floege J, Coppo R (2023). Evaluating progression risk in patients with immunoglobulin a nephropathy. Kidney International Reports.

[ref-7] Chen D, Liu J, Duan S, Chen P, Tang L, Zhang L, Feng Z, Cai G, Wu J, Chen X (2018). Clinicopathological features to predict progression of IgA nephropathy with mild proteinuria. Kidney and Blood Pressure Research.

[ref-8] Chen C-H, Wu M-J, Tsai S-F (2023). Validating the association of Oxford classification and renal function deterioration among Taiwanese individuals with Immunoglobulin A nephropathy. Scientific Reports.

[ref-9] Coppo R, D’Arrigo G, Tripepi G, Russo ML, Roberts ISD, Bellur S, Cattran D, Cook TH, Feehally J, Tesar V, Maixnerova D, Peruzzi L, Amore A, Lundberg S, Di Palma AM, Gesualdo L, Emma F, Rollino C, Praga M, Biancone L, Pani A, Feriozzi S, Polci R, Barratt J, Del Vecchio L, Locatelli F, Pierucci A, Caliskan Y, Perkowska-Ptasinska A, Durlik M, Moggia E, Ballarin JC, Wetzels JFM, Goumenos D, Papasotiriou M, Galesic K, Toric L, Papagianni A, Stangou M, Benozzi L, Cusinato S, Berg U, Topaloglu R, Maggio M, Ots-Rosenberg M, D’Amico M, Geddes C, Balafa O, Quaglia M, Cravero R, Lino Cirami C, Fellstrom B, Floege J, Egido J, Mallamaci F, Zoccali C (2020). Is there long-term value of pathology scoring in immunoglobulin A nephropathy? A validation study of the Oxford Classification for IgA Nephropathy (VALIGA) update. Nephrology, Dialysis, Transplantation.

[ref-10] Coppo R, Troyanov S, Bellur S, Cattran D, Cook HT, Feehally J, Roberts ISD, Morando L, Camilla R, Tesar V, Lunberg S, Gesualdo L, Emma F, Rollino C, Amore A, Praga M, Feriozzi S, Segoloni G, Pani A, Cancarini G, Durlik M, Moggia E, Mazzucco G, Giannakakis C, Honsova E, Sundelin BB, Di Palma AM, Ferrario F, Gutierrez E, Asunis AM, Barratt J, Tardanico R, Perkowska-Ptasinska A (2014). Validation of the Oxford classification of IgA nephropathy in cohorts with different presentations and treatments. Kidney International.

[ref-11] Gadola L, Cabrera MJ, Garau M, Coitiño R, Aunchayna MH, Noboa O, Alvarez MA, Balardini S, Desiderio G, Dibello N, Ferreiro A, Giró S, Luzardo L, Maino A, Orihuela L, Ottati MG, Urrestarazú A (2023). Long-term follow-up of an IgA nephropathy cohort: outcomes and risk factors. Renal Failure.

[ref-12] Hou J-H, Zhu H-X, Zhou M-L, Le W-B, Zeng C-H, Liang S-S, Xu F, Liang D-D, Shao S-J, Liu Y, Liu Z-H (2018). Changes in the spectrum of kidney diseases: an analysis of 40 759 biopsy-proven cases from 2003 to 2014 in China. Kidney Disease (Basel).

[ref-13] Howie AJ, Lalayiannis AD (2023). Systematic review of the oxford classification of IgA nephropathy: reproducibility and prognostic value. Kidney360.

[ref-14] Kanbay M, Yildiz AB, Yavuz F, Covic A, Ortiz A, Siriopol D (2022). The role of body mass index on IgA nephropathy prognosis: a systematic review and meta-analysis. International Urology and Nephrology.

[ref-15] Kataoka H, Ohara M, Shibui K, Sato M, Suzuki T, Amemiya N, Watanabe Y, Honda K, Mochizuki T, Nitta K (2012). Overweight and obesity accelerate the progression of IgA nephropathy: prognostic utility of a combination of BMI and histopathological parameters. Clinical and Experimental Nephrology.

[ref-16] Lerma EV, Bensink ME, Thakker KM, Lieblich R, Bunke M, Rava A, Wang K, Murphy MV, Oliveri D, Amari DT, Cork DMW, Velez JCQ (2023). Impact of proteinuria and kidney function decline on health care costs and resource utilization in adults with IgA nephropathy in the United States: a retrospective analysis. Kidney Medicine.

[ref-17] Levey AS, Inker LA, Coresh J (2014). GFR estimation: from physiology to public health. American Journal of Kidney Diseases.

[ref-18] Schena FP, Anelli VW, Trotta J, Di Noia T, Manno C, Tripepi G, D’Arrigo G, Chesnaye NC, Russo ML, Stangou M, Papagianni A, Zoccali C, Tesar V, Coppo R (2021). Development and testing of an artificial intelligence tool for predicting end-stage kidney disease in patients with immunoglobulin A nephropathy. Kidney International.

[ref-19] Shimamoto M, Ohsawa I, Suzuki H, Hisada A, Nagamachi S, Honda D, Inoshita H, Shimizu Y, Horikoshi S, Tomino Y (2015). Impact of body mass index on progression of IgA nephropathy among japanese patients. Journal of Clinical Laboratory Analysis.

[ref-20] Stamellou E, Seikrit C, Tang SCW, Boor P, Tesař V, Floege J, Barratt J, Kramann R (2023). IgA nephropathy. Nature Reviews Disease Primers.

[ref-21] Tang X, Wen Q, Zhou Q, Yang Q, Chen W, Yu X (2023). Prognostic significance of the extent of tubulointerstitial lesions in patients with IgA nephropathy. International Urology and Nephrology.

[ref-22] Thompson A, Carroll K, Inker LA, Floege J, Perkovic V, Boyer-Suavet S, Major RW, Schimpf JI, Barratt J, Cattran DC, Gillespie BS, Kausz A, Mercer AW, Reich HN, Rovin BH, West M, Nachman PH (2019). Proteinuria reduction as a surrogate end point in trials of IgA nephropathy. Clinical Journal of the American Society of Nephrology.

[ref-23] Trimarchi H, Barratt J, Cattran DC, Cook HT, Coppo R, Haas M, Liu Z-H, Roberts ISD, Yuzawa Y, Zhang H, Feehally J (2017). Oxford classification of IgA nephropathy 2016: an update from the IgA nephropathy classification working group. Kidney International.

[ref-24] Wang S, Qin A, Dong L, Tan J, Zhou X, Qin W (2023). Association of obesity with the development of end stage renal disease in IgA nephropathy patients. Frontiers in Endocrinology (Lausanne).

[ref-25] Wu C, Wang AY, Li G, Wang L (2018). Association of high body mass index with development of interstitial fibrosis in patients with IgA nephropathy. BMC Nephrology.

[ref-26] Yun H-R, Kim H, Park JT, Chang TI, Yoo T-H, Kang S-W, Choi KH, Sung S, Kim SW, Lee J, Oh K-H, Ahn C, Han SH (2018). Obesity, metabolic and abnormality, and progression of CKD. American Journal of Kidney Diseases.

